# Unraveling the Contribution of Serotonergic Polymorphisms, Prefrontal Alpha Asymmetry, and Individual Alpha Peak Frequency to the Emotion-Related Impulsivity Endophenotype

**DOI:** 10.1007/s12035-022-02957-6

**Published:** 2022-07-19

**Authors:** Florian Javelle, Andreas Löw, Wilhelm Bloch, Thomas Hosang, Thomas Jacobsen, Sheri L. Johnson, Alexander Schenk, Philipp Zimmer

**Affiliations:** 1 Clinical Exercise-NeuroImmunology Group, Department for Molecular and Cellular Sports Medicine, Institute for Cardiovascular Research and Sports Medicine, German Sport University Cologne, Cologne, Germany; 2 Experimental Psychology Unit, Helmut Schmidt University/University of the Federal Armed Forces Hamburg, Hamburg, Germany; 3 Department of Psychology, University of California Berkeley, Berkeley, CA USA; 4 Division of Performance and Health (Sports Medicine), Institute for Sport and Sport Science, Technical University Dortmund, Dortmund, Germany

**Keywords:** 5-HTTLPR, MAO-A, STin2, Emotion-related impulsivity, Individual alpha peak frequency, Alpha asymmetry

## Abstract

The unique contribution of the serotonin transporter-linked polymorphic region (5-HTTLPR), intronic region 2 (STin2), and monoamine oxidase A (MAO-A) genes to individual differences in personality traits has been widely explored, and research has shown that certain forms of these polymorphisms relate to impulsivity and impulsivity-related disorders. Humans showing these traits are also described as having an asymmetrical prefrontal cortical activity when compared to others. In this explorative study, we examine the relationship between serotonergic neurotransmission polymorphisms, cortical activity features (prefrontal alpha asymmetry, individual alpha peak frequency [iAPF]), emotion-related and non-emotion-related impulsivity in humans. 5-HTTLPR, MAO-A, and STin2 polymorphisms were assessed in blood taken from 91 participants with high emotion-related impulsivity levels. Sixty-seven participants completed resting electroencephalography and a more comprehensive impulsivity index. In univariate analyses, iAPF correlated with both forms of emotion-related impulsivity. In multiple linear regression models, 5-HTTLPR polymorphism (model 1, adj. *R*^2^ = 15.2%) and iAPF were significant interacting predictors of emotion-related impulsivity, explaining a large share of the results’ variance (model 2, adj. *R*^2^ = 21.2%). Carriers of the low transcriptional activity 5-HTTPLR and MAO-A phenotypes obtained higher emotion-related impulsivity scores than others did. No significant results were detected for non-emotion-related impulsivity or for a form of emotion-related impulsivity involving cognitive/motivational reactivity to emotion. Our findings support an endophenotypic approach to impulsivity, showing that tri-allelic 5-HTTLPR polymorphism, iAPF, and their interaction are relevant predictors of one form of emotion-related impulsivity.

**Supplementary Information:**

The online version contains supplementary material available at 10.1007/s12035-022-02957-6.

## Introduction

Impulsivity is a multidimensional personality construct associated with a wide range of psychological disorders (e.g., major depressive disorder, bipolar disorder, attention-deficit hyperactivity disorder, suicide) [1–5]. While older impulsivity definitions tended to focus on problems with planning, deliberation, and attention [6, 7], newer research reports the importance of impulsivity in response to states of high positive or negative emotions [2, 8, 9]. Emotion-related impulsivity has been defined as the reflexive tendency to act impulsively during periods of heightened emotion [9]. A large body of work shows that emotion-related impulsivity is more robustly tied to psychopathologies, aggression, and suicide than are other forms of impulsivity [10, 11].

**Fig. 1:**
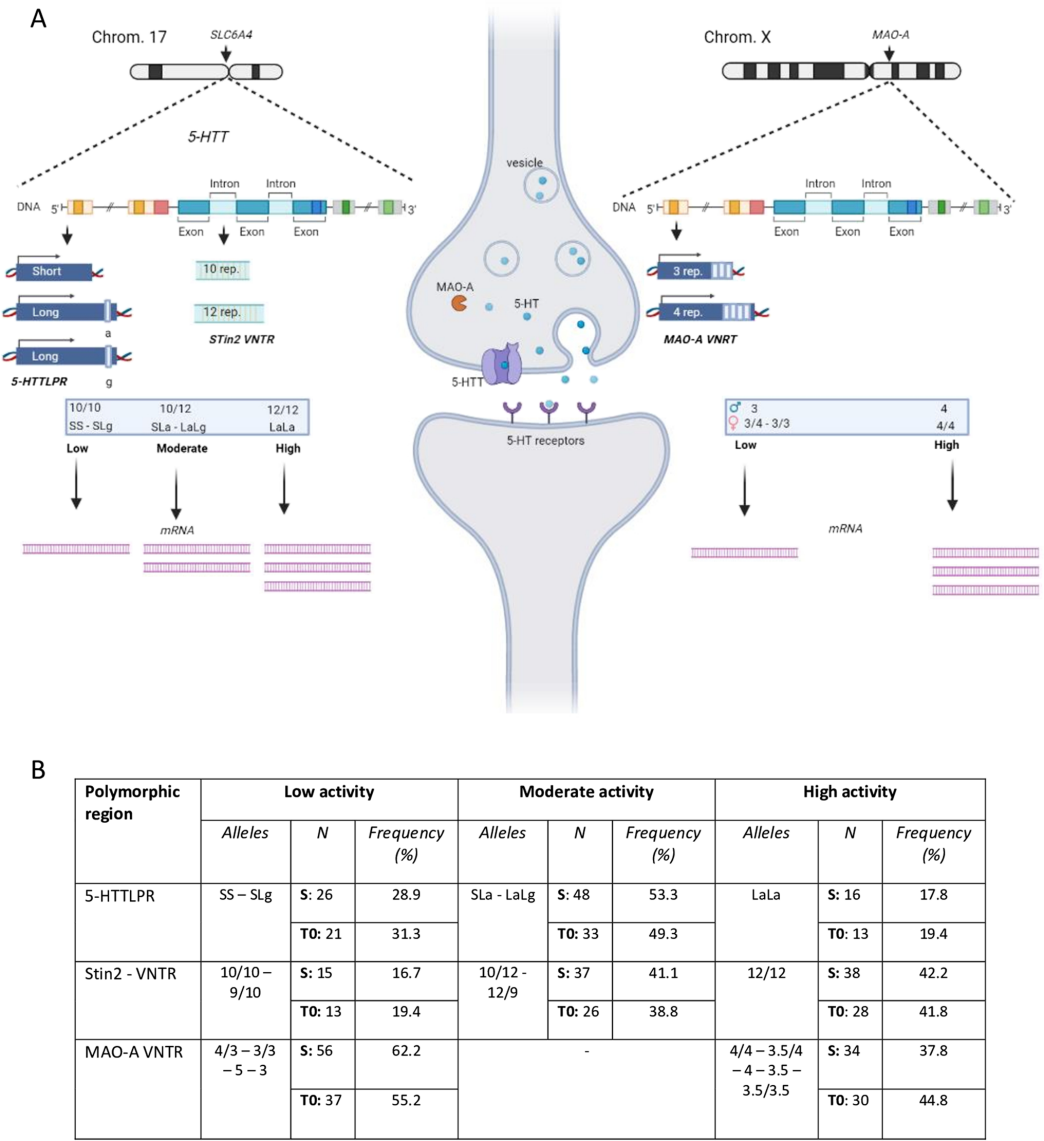
Allelic variations for serotonin transporter linked polymorphic region (5-HTTLPR), serotonin transporter intronic region 2 (STin2), and monoamine oxidase A (MAO-A) leading to different transcriptional activity phenotypes. Only the most frequent alleles are illustrated in **A**. Chrom., chromosome; 5-HTT, serotonin transporter; 5-HT, serotonin; rep., repeats; VNTR, variable number tandem repeat. This figure has been created using BioRender.com. **B** Phenotype categorization used in this study based on genes’ transcriptional activity levels and their occurrence frequency (in percentage) in this analysis. MAO-A being located on chromosome X, male participants comport only one allele of the gene. All results were concordant with participants’ gender. The phenotype categorization was realized using the following references: [14, 15, 17, 18]. S, short; L, long; a, adenine; g, guanine

Serotonergic neurotransmission modulates mood and emotion [12, 13], consequently affecting a wide spectrum of impulsivity-related traits. Two key regulators of serotonergic signaling are the serotonin transporter (5-HTT) and the monoamine oxidase A (MAO-A), which respectively remove serotonin from the synaptic cleft and catabolize monoamines with a strong affinity for serotonin and catecholamines [13–15]. The 5-HTT protein is encoded by the gene SLC6A4, whose transcriptional activity is modulated by several variations, including two repetitive sequences called the serotonin transporter-linked polymorphic region (5-HTTLPR) and the serotonin transporter intronic region 2 (STin2) variable number tandem repeat (VNTR) [16]. Likewise, the MAO-A gene has a VNTR polymorphism modulating its transcription. Transcriptional activity levels in each of these monoamine genes result in different expression rates of their respective mRNA and subsequent proteins and can therefore be classified in phenotypic categories (Fig. 1) [14, 15, 17, 18]. These phenotypic distinctions are also related to some psychological traits, especially impulsivity [14, 15].

More specifically, these polymorphisms have been associated with impulsivity and impulsivity-related disorders. For example, impulsivity [9], neuroticism [14], anxiety [19], and emotional instability [14] are more frequent in people having the low 5-HTTLPR transcriptional activity (5-HTTLPR_Low_) phenotype. The shortest repetitive sequences of STin2 are associated with elevated cognitive impulsivity [20], anxiety scores [21], major depressive disorders [22], early-onset bipolar disorder [23], and suicide attempts [24]. Finally, MAO-A phenotypes are linked to antisocial behaviors [15], emotional instability [25], impulsivity [26], bipolar disorders [27], and violent aggression [28].

Attempts to understand the neurogenetic basis of impulsivity have been limited by the relative absence of attention to the multidimensional nature of impulsivity. One previous study suggested that emotion-related impulsivity, but not non-emotion-related impulsivity, was tied to 5-HTTLPR [9]. Therefore, the aim of this explorative study was to evaluate serotonergic genetic markers while differentiating between emotion-related and non-emotion-related impulsivity.

Beyond these genetic markers, we consider other stable genetically modulated variables to provide a more comprehensive understanding of the determinants of impulsivity. Cortical activity, including lateralization of frontal oscillations in the alpha frequency band, is a partially genetically modulated parameter sometimes found to be associated with genetic polymorphisms such as the 5-HTTLPR_Low_ phenotype [29]. For more than 50 years, research has demonstrated that frontocortical regions are asymmetrically related to motivational and emotional variables, such as approach and avoidance tendencies: heightened relative activity in the left frontal cortex is related to approach motivation, whereas heightened relative activity in the right frontal cortex is related to avoidance motivation [30–33]. Impulsivity (in particular, its urgency and positive urgency dimensions) can be depicted as the inability to inhibit approach urges [34, 35]. Drawing on these findings, multiple investigators have shown that greater left prefrontal activity during rest (i.e., a right-sided predominance of alpha power as alpha oscillations are inversely related to cortical activity [36]) is associated with impulsivity [37–39].

There is reason to believe that emotion-related impulsivity may be more closely tied to lateralization indices than non-emotion-related impulsivity is. High emotional instability has frequently been associated with lateralization of the frontal alpha activity [29, 37–40]. Regions of the left prefrontal cortex are believed to play an important role in inhibiting the amygdala [41, 42], and their activation leads to downregulation of the amygdala when participants are asked to downregulate negative affect [43, 44]. Accordingly, we hypothesize that persons exhibiting highly impulsive responses to emotion may show stronger right alpha prefrontal activity than others.

Other cortical activity characteristics, such as individual alpha peak frequency (iAPF), are highly heritable, appear to be under substantial genetic control [45–47], and show high stability over test–retest intervals in healthy and clinical conditions [48, 49]. Given this, iAPF is considered to be a valuable marker for understanding psychological traits [50]. Furthermore, seminal research has already shown that alpha power and alpha peak frequency were higher in highly impulsive individuals when compared to low impulsive individuals [51]. Compared to the alpha power, the iAPF has more robust heritability estimates and better test–retest reliability [48, 52].

Thus, in the current study, we used an endophenotypic approach, combining genetic and cortical features to better understand the mechanisms underlying emotion-related impulsivity. Serotonergic neurotransmission polymorphisms (i.e., 5-HTTLPR, STin2, and MAO-A), prefrontal alpha asymmetry, and iAPF were assessed in emotionally impulsive humans. We hypothesized that the low transcriptional activity phenotypes in serotonergic neurotransmission polymorphisms and high right prefrontal alpha asymmetry would relate to higher emotion-related impulsivity. According to seminal research, the iAPF should be higher in highly impulsive individuals when compared to others.

## Material and Methods

All samples and data used in this explorative study were taken at the baseline testing of an intervention study called NoSTRESS*.* The authors assert that all procedures contributing to this work comply with the ethical standards of the relevant national and institutional committees on human experimentation and with the Helsinki Declaration of 1975, as revised in 2008. The study was registered on the German Clinical Trial registration website (DRKS00016589) and approved by the university review board before data collection.

### Participants

**Fig. 2:**
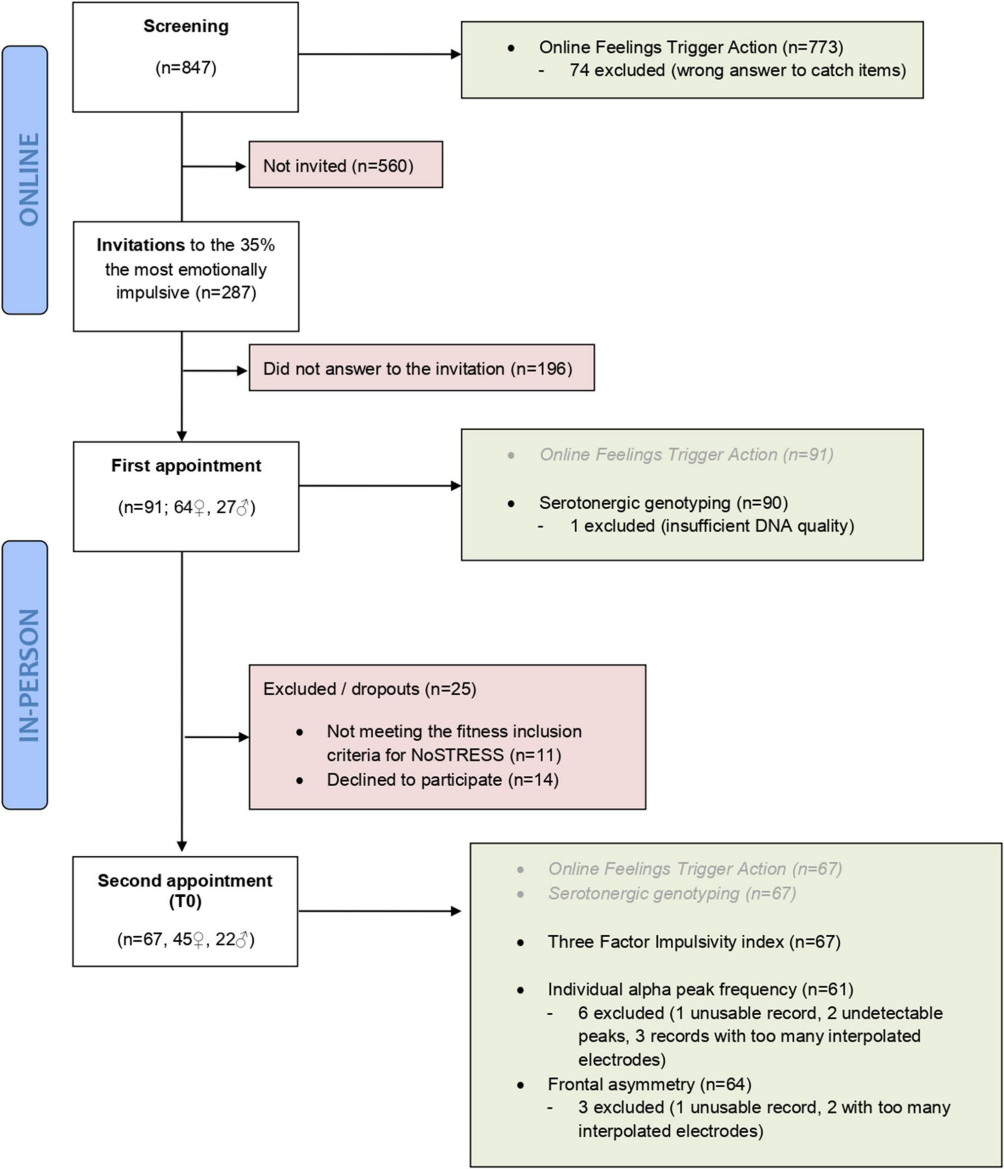
Flow diagram of the study. Green boxes refer to valid data available at each stage of the study. In grey are recalled valid data that were previously gathered. Red boxes refer to excluded or dropout participants

Participants were recruited through advertising on the university website and online social networks (i.e., Facebook, Twitter, and LinkedIn) based on high scores on a measure of emotion-related impulsivity. As presented in Fig. 2, 91 participants (one participant later excluded because of insufficient DNA quality) were included in genotyping step (mean age = 29.9 ± 7.7). Sixty-seven participants attended a second appointment where the resting electroencephalogram (EEG) and fuller impulsivity measurements were gathered (mean age = 30.3 ± 7.6). Details are presented in Fig. 2.

### Experimental Design

Participants completed consent, a self-report measure of emotion-related impulsivity (Feelings Trigger Action) [9], and questions on inclusion/exclusion criteria via the Qualtrics platform. The 35% of participants with the highest emotion-related impulsivity levels were invited to the laboratory to perform a quasi-ramp cardiopulmonary exercise testing (CPET; not relevant here but available in [53]) and blood testing between 7 and 11 a.m. and in a fasted state (8–16 h).

The exclusion and inclusion criteria were double-checked at the lab, and written informed consent was gathered. A short medical check-up was conducted. Blood was drawn via venipuncture at rest. On a separate day within the same week, participants were invited to a second appointment to record resting cortical activity and complete a broader index of impulsivity, the Three-Factor Impulsivity index [9].

### Inclusion/Exclusion Criteria

Exclusion criteria were designed for the NoSTRESS study [53] and included current pregnancy, breastfeeding, specific medical conditions or psychological disorders diagnosed by clinicians, use of antidepressant medication, engagement in more than three hours of exercise per week, and fitness level (i.e., VO_2_peak) at or below fair (age and gender corrected, based on the Standards of the Federal Office for Sport [Bundesamtes für Sport]). Participants had to be native German speakers, between 18 and 50 years old, and among the 35% highest emotion-related impulsivity scores [53] (DRKS00016589).

### DNA Isolation

Blood samples were taken in K2 EDTA tubes, and buffy coats were collected after two centrifugation cycles—phosphate-buffered saline (PBS) re-suspension. Freezing medium was added to the samples (1 mL for 300 µL of buffy coat) that were stored at − 150 °C until study completion. On the day of analysis, the buffy coat was brought to room temperature and then centrifuged for 10 min at 5500 rpm. The supernatant was discarded. The pellet was re-suspended with a 200 µL PBS solution. DNA was isolated using Blood DNA Mini Kit (Bio-Budget, Technologies GmbH, Germany). Quantification and quality assessment of the DNA were performed using a single sample spectrophotometer (NanoDrop 1000, peqlab, biotechnologie, GmbH). Samples were stored at − 20 °C until required.

### Genotyping

Primers were synthesized by Invitrogen, Thermo Fisher Scientific, Germany (Supplementary Material A) based on former uses [54–56]. Participant’s genomic DNA (20 ng) was amplified using the premixed ready-to-use solution GoTaq Colorless MasterMix (Promega, Madison, USA) and equimolar concentrations (50 pmol final concentration) of forward and reverse primers for 5-HTTLPR, STin2, and MAO-A (Invitrogen, Thermo Fisher Scientific, Germany). Complete polymerase chain reaction (PCR) cycling conditions for each gene are described in Supplementary Material A. Annealing temperatures were determined using temperature gradient (63° for MAO-A and STin2, 61° for 5-HTTLPR). To determine the presence of the rs25531 single nucleotide polymorphism within the 5-HTTLPR region, a restriction enzyme digest was performed on the PCR amplicons. Twenty microliters of the product was digested with 1 µL MspI (New England Biolabs, Herts, UK) at 37 °C for 6 h. PCR amplicons were separated by 0.5 × tris–borate-EDTA-buffered 1.5% agarose gel (loading dye without sodium dodecyl sulfate) and visualized with ethidium bromide using a UV trans-illuminator. All samples were duplicated for assay reliability. Interpretation of the PCR products are presented in Supplementary Material A. Phenotype and genotype occurrences are presented, respectively, in Fig. 1B and Supplementary Material A.

### Three-Factor Impulsivity Index [9]

The Three-Factor Impulsivity index is a 54-item composite self-report measure of impulsivity. Items are rated from 1 (“I strongly disagree”) to 5 (“I strongly agree”), with higher scores reflecting higher impulsivity levels. The questionnaire covers eight different components of impulsivity shown in oblique factor analyses and confirmatory structural equation modeling to load onto three separate factors [9, 57]: Pervasive Influence of Feelings, (lack of) Follow-Through, and Feelings Trigger Action; alphas = 0.837, 0.897, and 0.857, respectively. The first and the third factors are emotion-related. Factor one, Pervasive Influence of Feelings, reflects unconstrained cognitive and motivational responses to negative emotions and includes items from previously validated scales for negative generalization [58], negative urgency [8], and novel items to capture lethargy in response to sadness and extremely negative thoughts of self and the world in response to emotions. Factor two, (lack of) Follow-Through, includes items from previously validated scales designed to cover distractibility and lack of perseverance [8]. The third factor, Feelings Trigger Action, which was used for screening, includes items from the previously validated scales of Negative Urgency [8], Positive Urgency [59], and items to capture responding reflexively and quickly when experiencing emotions. The German validated version of the questionnaire was used [60]. One attention catch item (“Please select I agree”) was embedded in the online screening questionnaire, and two such items in the full Three-Factor Impulsivity index. All participants answered the three catch items properly, and no data were removed for failing these items. Means and standard deviation were 3.19 ± 0.71 for Pervasive Influence of Feelings (*n* = 67), 2.80 ± 0.59 for (lack of) Follow-Through (*n* = 67), 3.11 ± 0.44 for Feelings Trigger Action at T0 (*n* = 67), and 3.22 ± 0.35 for Feeling Trigger Action at screening (*n* = 91).

### Electroencephalography

#### Data Acquisition

The EEG was recorded continuously for 5 min while participants sat alone in a relaxed position with eyes closed in a testing room with temperature and humidity maintained at 20.5 ± 0.5 °C and 44 ± 11%, respectively. A BioSemi Active-Two system (BioSemi, Amsterdam, Netherlands) with 64 Ag/AgCl electrodes embedded in an elastic cap and placed according to the international 10–20 system was used. The system records the voltage between each electrode and an active common mode sense (CMS) electrode that forms a feedback loop with a passive drive right leg (DLR) electrode. CMS and DLR were located in parieto-occipital positions. The sampling frequency was set at 2048 Hz, and the electrodes’ offset was kept below 50 µV. ActiView software (BioSemi, Amsterdam, Netherlands) was used to record the data.

#### Data Analysis

Offline EEG data processing was conducted using Python’s (v3.8.5) MNE package (v0.22.0) [61]. First, power line noise at 50 Hz and its respective harmonics was attenuated by notch filters (overlap-add finite impulse response filtering). Then, the data were re-referenced to the average reference. Bad channels were detected automatically using the noisy channel detection algorithm of pyprep (per deviation; threshold = 5z) [62] and via visual inspection. If bad channels were detected, they were subsequently removed, and data were interpolated using spherical splines as long as three original neighboring signals were available for interpolation. Muscle artifacts were automatically detected and annotated within the continuous raw data using the MNE annotate_muscle_zscore method. Then, the raw data were filtered with a 1-Hz high-pass and 40-Hz low-pass filter (both overlap-add finite impulse response filtering). Power spectra density was computed by Welch’s method using 1-s segments with 50% overlap. Segments containing previously annotated muscle artifacts and a peak-to-peak amplitude exceeding 200 µV in any channel were rejected. On average across participants, 533 ± 47 quality-sufficient epochs were used in the analysis.

Scientific literature provides a spectrum of studies linking impulsivity to different frontal cortex regions, e.g., [31–33, 37, 39, 63–65]. Thus, we used a comprehensive approach going from the general to the specific. In preliminary analyses, we have first tested the frontal cortex, then the prefrontal cortex, and finally specific pairs of electrodes from the prefrontal region often used in asymmetry literature: Fp2/Fp1, F2/F1, F4/F3, and F8/F7 (details in Supplementary Material A). These multiple tests were adjusted using a family-wise Bonferroni correction (see “Statistics” section). As suggested in previous research [29], the alpha asymmetry was indexed using laterality coefficients (LC) using the following formula: LC = (power right – power left) / (power right + power left) × 100. Values superior to zero indicate higher alpha activity in the right cortex compared to the left one, in other words, a greater left cortical activity. LC has been used for a long time in the field of laterality because it separates the asymmetry variance from the general magnitude variance [64]. This score is perfectly correlated with another metric commonly reported in EEG studies (ln (right) – ln (left)) [66]. Nevertheless, using the LC allows easier comparison between different studies, different frequency bands, and locations [67].

IAPF was estimated from the power spectra densities computed from 1-s non-overlapping segments that were zero-padded to 10 s to have a frequency resolution of 0.1 Hz. The iAPF between 8 and 13 Hz was determined from the mean over 17 posterior electrodes (Pz, P1/2, P3/4, P5/6, P7/8, POz, PO3/4, PO7/8, Oz, and O1/2) [50].

### Statistics

Statistical analyses were conducted using R (v1.2.1335) and SPSS (v23). The dataset and the R script are provided in Supplementary Materials B and C. Data were first *z*-standardized (for dependant variables and continuous predictors) and then winsorized at ± 3z [68]. The distribution of each variable was evaluated using the Shapiro–Wilk test and checked for linearity (via quantile–quantile plots and histograms of standardized residuals), skewness, and kurtosis. Pearson’s bivariate correlations were used to evaluate the relationship of EEG markers with impulsivity levels. As the alpha asymmetry was evaluated using six parameters, a Bonferroni alpha correction was applied family-wise by setting the significance level at *p* < 0.008. All univariate analyses were controlled for age and gender.

Multiple linear regression models were used to assess how gene phenotypes, individual alpha frequency, and cortical asymmetry LC contributed to impulsivity (continuous scores). The predictors were either categorical (i.e., phenotypes, gender) or continuous (i.e., iAPF, LCs, age) [68]. As the MAO-A gene is located on the X chromosome, and impulsivity can vary with gender, gender was included as a potential predictor. Because impulsivity and iAPF have been shown to change with age, age was included as a predictor [50, 69]. To avoid potential collinearity, we included F4/F3, the most commonly used asymmetry marker, as the only pre-frontal-asymmetry marker. Based on the literature, one can expect interaction between 5-HTTLPR polymorphisms and cortical activity [29]. Thus, these interaction terms were included in the regression models. Missing values were imputed based on the mean when inferior to 10% of the total number of values (F4/F3 LC: 4.5% and iAPF: 8.9%) [70, 71].

All predictors were checked for multicollinearity (variance inflation factor and tolerance values were acceptable at < 2 and > 0.2, respectively), independence (Durbin Watson test was acceptable, results ranged from 1 to 3), and linearity (graphically via quantile–quantile plot, scatterplot, and histogram of studentized residuals) [72]. All assumptions for the statistical analysis were met. As models 1 and 2 both assessed Feeling Trigger Action, a Bonferroni alpha correction was applied, bringing the significance level to *p* < 0.025. For the other parameters, the significance level was set to *p* < 0.050.

## Results

### Univariate Analyses

#### Impulsivity and Polymorphisms

Average impulsivity scores per gene and transcriptional activity phenotypes are reported in Supplementary Material A. When controlled for age and gender, Feelings Trigger Action levels at screening differed significantly among 5-HTTLPR transcriptional activity phenotypes *F*(2, 85) = 8.853, *p* < 0.001, *np*^2^ = 0.172 and at T0 *F*(2, 62) = 3.220, *p* < 0.050, *np*^2^ = 0.094. Post hoc analyses revealed that carriers of the 5-HTTLPR_Low_ phenotype had significantly higher Feelings Trigger Action scores than the moderate (5HTTLPR_Moderate_) and high transcriptional activities (5-HTTLPR_High_) phenotype carriers. Moreover, Feelings Trigger Action scores were significantly higher for the low MAO-A VNTR transcriptional activity (MAO-A_Low_) phenotype carriers compared to the high ones (MAO-A_High_), *F*(1, 63) = 4.927, *p* < 0.050, *np*^2^ = 0.073 at T0, but were only a trend at screening, *F*(1, 86) = 3.620, *p* = 0.060, *np*^2^ = 0.040. No other phenotypes were significantly related to impulsivity scores (Supplementary Material A).

#### Impulsivity and EEG

**Fig. 3:**
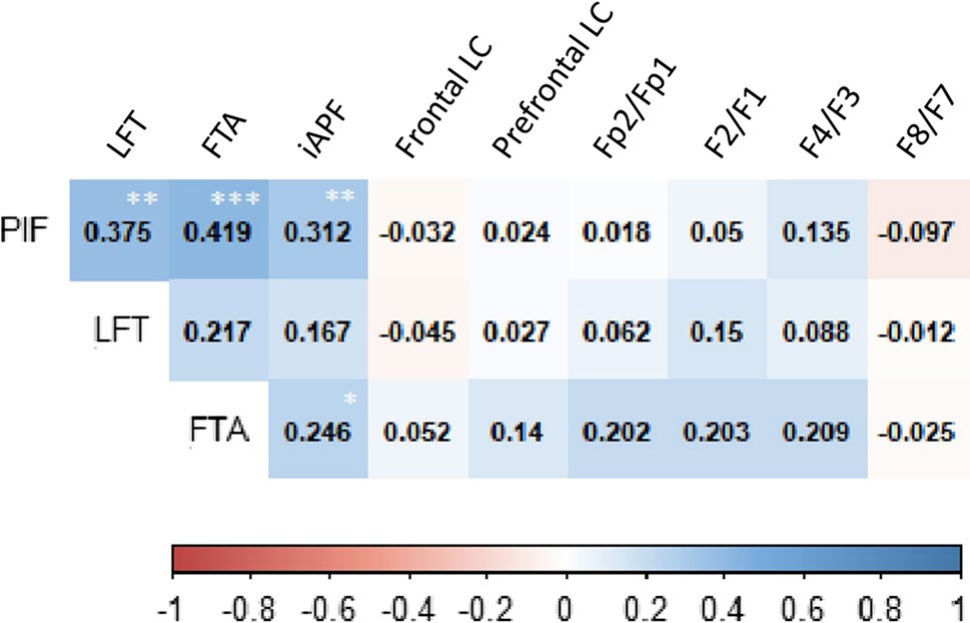
Pearson’s correlation coefficients of cortical activity markers and impulsivity scores (T0). PIF, Pervasive Influence of Feelings; LFT, (Lack of) Follow-Through; FTA, Feelings Trigger Action; LC, Laterality Coefficient; iAPF, individual alpha peak frequency. Significance for the asymmetry: #*p* < 0.008. For the rest: *p < 0.050; **p < 0.010; ***p < 0.001

When cortical activity markers were correlated to impulsivity scores, iAPF was positively correlated to both emotion-related impulsivity factors, *| r's |*> 0.246, *p's* < 0.050 (Fig. 3). No other significant results for cortical activity makers with impulsivity were detected. Nevertheless, as shown in Fig. 3, non-significant trends between three binary prefrontal asymmetry LCs (Fp2/Fp1, F2/F1, F4/F3) were observed, 0.113 > *p's* > 0.092. Age was not correlated with any EEG markers.

### Multiple Linear Regression Models

Multiple linear regression models are reported in Table 1 for Feelings Trigger Action at screening (*n* = 90), and for a smaller sample, all three impulsivity scales at T0 (*n* = 67). Of the four models, only the two with Feelings Trigger Action were significant. Model 1 explained 21.9% (adj. *R*^2^ = 15.2%) of the total variance of Feelings Trigger Action at screening (*p* < 0.010). Out of the five predictors included in the Feelings Trigger Action screening model, only 5-HTTLPR and MAO-A polymorphisms were significant. Beta scores (Table 1) show that carriers of 5-HTTLPR_High_ and MAO-A_High_ phenotypes have lower Feelings Trigger Action scores when compared to the low transcriptional activity phenotypes. When standardized, the 5-HTTLPR beta scores had the strongest magnitude (Table 1). When iAPF, F4/F3 LC, and their interaction terms with 5-HTTLPR were added, the second model explained 36.7% (adj. *R*^2^ = 21.2%) of the total variance (*p* < 0.025). Out of the nine predictors, 5-HTTLPR_Moderate_ , iAPF, and their interaction term were significant (Table 1). When compared to 5-HTTLPR_Low_ phenotype, 5-HTTLPR_Moderate_ carriers had lower Feelings Trigger Action scores. On the contrary, Feelings Trigger Action scores conjointly increased with iAPF. Nevertheless, the interaction term 5-HTTLPR_Moderate _/ iAPF had a negative beta score, indicating an inverse relation. Even though not significant, the interaction term 5-HTTLPR_High _/ iAPF also had a negative beta score suggesting that the positive relation between iAPF and Feeling Trigger Action scores was very likely to be driven by 5-HTTLPR_Low_ phenotypic category*.* Even though models 3 and 4 explained an important part of variance (respectively, 19.3% and 25.4%), neither was significant (Table 1).

**Table 1:** Multiple linear regression models

Model	*R*^2^- *p* value	Variable	Standardized estimate (std. β)	Estimate (*β*)	Std. error	*p* value
1 Feelings Trigger Action – screening	Multiple *R*^2^ = 21.9% Adj. *R*^2^ = 15.2% *p* < 0.010	**Intercept**	**0**	**0.803**	**0.265**	** < 0.001**
		**5-HTTLPR**_**Moderate**_	**-0.423**	**-0.856**	**0.234**	** < 0.001**
		**5-HTTLPR**_**High**_	**-0.359**	**-0.933**	**0.299**	** < 0.001**
		STin2_Moderate_	-0.061	-0.122	0.297	0.681
		STin2_High_	-0.047	-0.095	0.293	0.746
		**MAO-A**_**High**_	**-0.212**	**-0.431**	**0.213**	** < 0.050**
		Gender_Male_	0.124	0.270	0.231	0.246
		Age	0.104	0.104	0.107	0.333
2 Feelings Trigger Action – T0	Multiple *R*^2^ = 36.7% Adj. *R*^2^ = 21.2% *p* < 0.025	Intercept	0	0.407	0.311	0.197
		**5-HTTLPR**_**Moderate**_	**-0.286**	**-0.567**	**0.257**	** < 0.050**
		5-HTTLPR_High_	-0.230	-0.577	0.332	0.088
		STin2_Moderate_	-0.040	-0.081	0.344	0.814
		STin2_High_	-0.014	-0.028	0.333	0.934
		MAO-A_High_	-0.190	-0.333	0.245	0.128
		**iAPF**	**0.601**	**0.601**	**0.189**	** < 0.010**
		F4/F3 score	0.301	0.303	0.239	0.211
		**5-HTTLPR**_**Moderate**_**/iAPF**	**-0.406**	**-0.649**	**0.264**	** < 0.050**
		5-HTTLPR_High_/iAPF	-0.219	-0.560	0.361	0.127
		5-HTTLPR_Moderate_/F4/F3 score	0.022	0.029	0.282	0.918
		5-HTTLPR_High_/F4/F3 score	-0.183	-0.439	0.373	0.244
		Gender_Male_	0.104	0.220	0.280	0.436
		Age	0.222	0.224	0.137	0.108
3 Pervasive Influence of Feelings - T0	Multiple *R*^2^** = **19.3% Adj. *R*^2^** = **0% *p* = 0.487	Intercept	0	-0.544	0.647	0.404
		5-HTTLPR_Moderate_	-0.122	-0.242	0.290	0.407
		5-HTTLPR_High_	-0.008	-0.020	0.375	0.959
		STin2_Moderate_	0.013	0.026	0.388	0.946
		STin2_High_	-0.020	-0.040	0.376	0.917
		MAO-A_High_	-0.071	-0.141	0.277	0.613
		iAPF	0.251	0.251	0.213	0.243
		F4/F3 score	0.322	0.324	0.270	0.236
		5-HTTLPR_Moderate_/iAPF	0.025	0.040	0.298	0.894
		5-HTTLPR_High_/iAPF	0.143	0.367	0.408	0.372
		5-HTTLPR_Moderate_/F4/F3 score	-0.152	-0.202	0.319	0.529
		5-HTTLPR_High_/F4/F3 score	-0.077	-0.185	0.421	0.662
		Gender_Male_	-0.143	-0.301	0.316	0.344
		Age	0.213	0.028	0.020	0.170
4 Lack of Follow-Through – T0	Multiple *R*^2^** = **25.4% Adj. *R*^2^** = **7.0% *p* = 0.200	Intercept	0	0.893	0.622	0.157
		5-HTTLPR_Moderate_	-0.137	-0.273	0.279	0.332
		5-HTTLPR_High_	0.016	0.041	0.361	0.910
		STin2_Moderate_	-0.094	-0.190	0.373	0.612
		STin2_High_	-0.260	-0.523	0.362	0.154
		MAO-A_High_	0.163	0.326	0.267	0.227
		iAPF	0.403	0.403	0.205	0.054
		F4/F3 score	0.400	0.402	0.260	0.128
		5-HTTLPR_Moderate_/iAPF	-0.194	-0.310	0.286	0.285
		5-HTTLPR_High_/iAPF	-0.083	-0.213	0.392	0.589
		5-HTTLPR_Moderate_/F4/F3 score	-0.194	-0.258	0.306	0.404
		5-HTTLPR_High_/F4/F3 score	-0.205	-0.492	0.405	0.230
		**Gender**_**Male**_	**0.289**	**0.610**	**0.304**	** < 0.050**
		Age	-0.021	-0.028	0.019	0.157

**Fig. 4:**
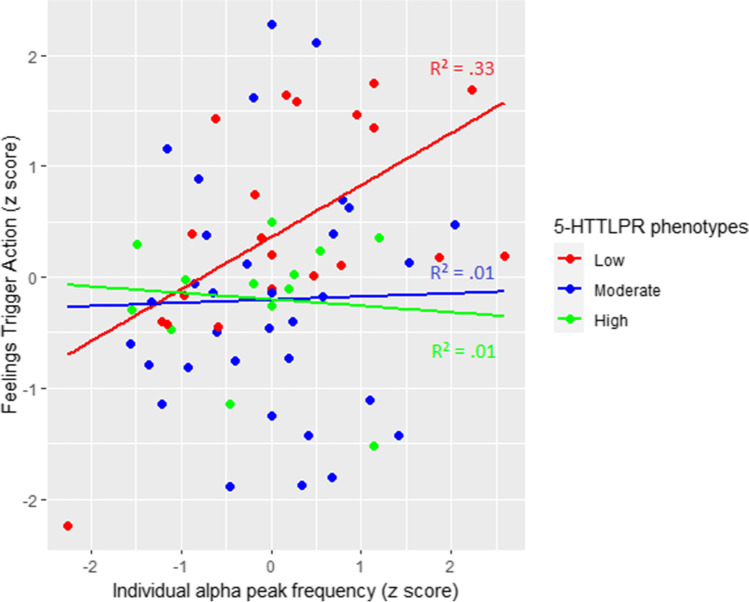
Multiple linear regression illustrations of Feelings Trigger Actionand its significant predictors: individual alpha peak frequency (iAPF) and serotonin transporter-linked polymorphic region (5-HTTLPR) phenotypes

Significant predictors of model 2 are displayed in Fig. 4. The graphic clearly illustrates the interaction between iAPF and 5-HTTLPR phenotypes, showing a strong positive link between iAPF and Feelings Trigger Action scores in participants with the 5-HTTLPR_Low_ phenotype.

## Discussion

Impulsivity triggered by positive or negative emotions has been shown to be more clinically relevant than other forms unrelated to emotions [73, 74]. Given the evidence that impulsivity is linked not only to serotonergic neurotransmission polymorphisms (i.e., 5-HTTLPR, STin2, and MAO-A) but also to cortical activity (i.e., iAPF and prefrontal alpha asymmetry), this explorative study aimed to assess the conjoint importance of these variables in explaining emotion-related impulsivity. The study examined two forms of emotion-related impulsivity— Feelings Trigger Action, which captures tendencies to engage in regrettable behavior in response to emotion, and Pervasive Influence of Feelings, which covers unconstrained cognitive/motivational responses to emotions. Both forms of emotion-related impulsivity were linked to higher iAPF. Carriers of MAO-A_Low_ and 5-HTTLPR_Low_ phenotypes had higher Feelings Trigger Action scores. Furthermore, findings of the multiple regression models show that the 5-HTTLPR polymorphism, iAPF, and their interaction term are significant predictors of Feelings Trigger Action scores. Significant effects of the 5-HTTLPR polymorphism were observed for both administrations of the Feelings Trigger Action subscale. These findings support the endophenotypic approach to emotion-related impulsivity.

In line with previous research [9], the moderate to large effect sizes detected between Feelings Trigger Action scores and 5-HTTLPR (at screening and T0) and MAO-A (T0) transcriptional activity phenotypes confirm the importance of these polymorphisms in explaining emotion-related impulsivity levels. This fits with the findings from several studies showing that carriers of 5-HTTLPR_Low_ and MAO-A_Low_ phenotypes show hyper-responsivity of the amygdala and anterior cingulate cortex during the display of negative emotional stimuli [75–78]. Even though it would be logical to relate the transcriptional activity levels of these genes to monoamine rates within neurons and synapses, research suggests that this relation is more complex. This reactivity is tied to decreased functional connectivity between the amygdala and anterior cingulate cortex in the 5-HTTLPR_Low_ , compared with 5-HTTLPR_High_ phenotype carriers [76]. The source of this functional connectivity difference might arise during fetal neurodevelopment, in that 5-HTTLPR_Low_ and MAO-A_Low_ phenotypes have, respectively, fewer 5-HTT and MAO-A mRNA levels in placenta tissue [56]. Therefore, emotion-related impulsivity levels could be driven by fetal monoamine levels affecting structural connectivity, and consequently functional interactions within neural circuits that regulate emotional reactivity.

We observed only non-significant trend-level correlations between prefrontal alpha asymmetry and emotion-related impulsivity. Prefrontal alpha asymmetry, though, is influenced not just by trait-like characteristics but also tends to shift in a state-dependent manner, and our current study design did not allow us to consider the state-like dynamic variation. We also did not record handedness preference or state levels of affect and motivation, which can potentially influence asymmetry results. Finally, post hoc power analyses (power > 0.8) suggest that effect sizes below *r* = 0.260 were probably undetectable.

Despite the null effect in the correlational analyses for alpha asymmetry, our findings suggest that a form of emotion-related impulsivity related to regrettable behavior (Feelings Trigger Action) is tied to a set of neural (iAPF) and genetic variables (5-HTTLPR and MAO-A). Intriguingly, results were not generalized to unconstrained cognitive or motivational responses to emotion (Pervasive Influence of Feelings), dovetailing with previous work to suggest the importance of distinguishing between these two factors in understanding neurocognitive correlates and psychopathology.

Univariate analyses indicated that the cognitive and behavioral forms of emotion-related impulsivity were both correlated to higher iAPF. These results are counterintuitive when considering research linking decreasing iAPF to central system pathologies [50] and high resting iAPF to high executive function performances [79]. Nonetheless, it is important to recall that these measurements were obtained from healthy participants without any emotional trigger. Furthermore, regression model 2 (Table 1) did put in evidence a significant interaction between the two predictors (i.e., iAPF and 5-HTTLPR), displaying a very different relationship between iAPF and Feelings Trigger Action scores per 5-HTTLPR phenotypes (Fig. 4). The positive relation depicted in the univariate analysis and in the beta score of iAPF (Table 1, model 2) appeared to be driven by the large effect in the carriers of the 5-HTTPLR_Low_ (*n* = 19, *R*^2^ = 0.330, *p* < 0.010). These ties were of negligible magnitude for 5-HTTLPR_Moderate_ (*n* = 30, *R*^2^ = 0.001, *p* = 0.900) and 5-HTTLPR_High_ (*n* = 11, *R*^2^ = 0.014, *p* = 0.726). More research investigating the link between 5-HTTLPR and iAPF is warranted. Moreover, 5-HTTLPR should now be considered systematically in iAPF research to have a better understanding of the results.

Despite the intriguing and novel findings, the results of this study should be interpreted within the context of several limitations. First, even though this study used a multifactorial endophenotypic approach, the scope of the investigation was limited. For example, polymorphisms in the dopaminergic pathway [80, 81], circulating levels of tryptophan [53, 82], central serotonin shortage [83, 84], and activity of the kynurenine pathway [53] have been tied to impulsivity but were not assessed in the present study. Second, the recruited sample had only a modest range of impulsivity scores (the 35% highest emotion-related impulsivity scores), which may have reduced result variance and constrained effect sizes. Third, even though most research in the field of alpha asymmetry underlying personality traits recommend resting EEG (e.g., [31, 32, 39, 63]), another body of research suggests that this condition might not be the most optimal [65, 85]. They argue that uncontrolled experimental conditions may affect resting measures and that individuals can engage in a variety of mental states that are not controlled during the resting tasks. As we used resting EEG condition, we cannot exclude that these parameters may have affected our results. Including an additional motivational induction paradigm followed by EEG measures [65, 85] in future studies may provide further insights into the relationship between frontal asymmetry and impulsivity. Finally, it is worth noting that future studies might also consider using clinical interviews and behavioral tests (e.g., Go/No-Go test) to gain deeper insights into impulsivity.

In conclusion, our results highlight the importance of using endophenotypic approaches to characterize impulsivity, demonstrating that the 5-HTTLPR polymorphism, iAPF, and their interaction are relevant predictors of a key form of emotion-related impulsivity involving regrettable behavior. Moreover, carriers of 5-HTTPLR_Low_ and MAO-A_Low_ phenotypes showed higher levels of this form of emotion-related impulsivity than did those with other phenotypes. This multifactorial neurogenetic approach to impulsivity could be applied to developing better identification and prediction for impulsivity-related disorders. Our findings were specific to emotion-related impulsivity involving regrettable behavior, which is of importance given the burgeoning literature suggesting that this form of impulsivity is uniquely powerful in predicting externalizing, suicidal behavior, and other key outcomes [1]. Evaluating conjoint changes between impulsivity and epigenetic mechanisms in serotonergic neurotransmission polymorphisms (e.g., DNA methylation, histone acetylation) represents another rich domain for future work that can be incorporated into endophenotypic approaches.

## Supplementary Information

Below is the link to the electronic supplementary material.

Supplementary file1 Supplementary Material A includes details about the genotyping methods and results. (DOCX 26 KB)

Supplementary file2 Supplementary Material B includes the dataset. (CSV 12 KB)

Supplementary file3 Supplementary Material C includes the R script. (R 16 KB)

## Data Availability

Data used in this analysis are provided in Supplementary Material B.

## Code Availability

The R code used to perform this analysis is provided in Supplementary Material C.
