# Supplementary material for: Unraveling the Contribution of Serotonergic Polymorphisms, Prefrontal Alpha Asymmetry, and Individual Alpha Peak Frequency to the Emotion-Related Impulsivity Endophenotype
Source: Mol Neurobiol. 2022 Jul 19;59(10):6062–75. doi: 10.1007/s12035-022-02957-6 (PMC9463349; doi:10.1007/s12035-022-02957-6)
Supplement: Supplementary file 1 — Supplementary file1 Supplementary Material A includes details about the genotyping methods and results. (DOCX 26 KB) [file 12035_2022_2957_MOESM1_ESM.docx]

1. **Genotyping methods and results**

Genes' primers are listed in Table 1.

Table 1

*Genes' primers****.***

| 5-HTTLPR & rs25531 | FWD: 5’-TCC TCC GCT TTG GCG CCT CTT CC-3' |
| --- | --- |
|  | REV: 5’-TGG GGG TTG CAG GGG AGA TCC TG-3' |
| MAO-A VNTR | FWD: 5′‐ACA GCC TGA CCG TGG AGA AG‐3′ |
|  | REV: 5′‐GAA CGG ACG CTC CAT TCG GA‐3′ |
| STin2 VNTR | FWD: 5’-GGG CAA TGT CTG GCG CTT CCC CTA CAT A-3' |
|  | REV: 5' -TTC TGG CCT CTC AAG AGG ACC TAC AGC-3' |

*FWD: Forward – REV: Reverse – VNTR: Variable Number Tandem Repeats – 5-HTTLPR: Serotonin Transporter-Linked Polymorphic Region*

All annealing temperatures were determined using a temperature gradient.

PCR conditions for 5-HTTLPR were an initial denaturalization of 5 min at 95°C, 32 cycles of 1min at 94°C, 1min at 61°C, and 1 min at 72°C; followed by a final elongation of 5 min at 72°C. PCR conditions for STin2 and MAO-A were an initial denaturalization of 2 min at 95°C, 30 cycles of 1min at 94°C, 1min at 63°C, and 1 min at 72°C; followed by a final elongation of 5 min at 72°C. PCR products were separated and visualized using 1.5% agarose gels with ethidium bromide.

Table 2

*PCR product sizes.*

| Genes | PCR products |
| --- | --- |
| 5-HTTLPR | Short (S) - 469bp  Long (L) - 512bp |
| 5-HTTLPR & rs25531 *(after MSPI digest)* | Sa - 469bp (uncut)  La - 512bp (uncut) Lg - 402bp + 110bp |
| MAO-A VNTR | 3 repeats - 350bp  3.5 repeats - 365  4 repeats - 380bp  5 repeats - 420 bp |
| STin2 VNTR | 9 repeats - 248bp  10 repeats - 265bp  12 repeats - 299bp |

*bp: base pairs - VNTR: Variable Number Tandem Repeats – 5-HTTLPR: Serotonin Transporter-Linked Polymorphic Region – a: adenine – g: guanine*

Table 3

*Participants’ genotypes frequency.*

| **Genes** | **Genotypes** | **Frequency** |
| --- | --- | --- |
| 5-HTTLPR and rs25531 | LaLg | 2 (2.2%) |
|  | SS | 23 (25.6%) |
|  | SLa | 46 (51.1%) |
|  | SLg | 3 (3.3%) |
|  | LaLa | 16 (17.8%) |
| STin2 VNTR | 12/12 | 38 (42.2%) |
|  | 12/10 | 35 (38.9%) |
|  | 10/10 | 13 (14.4%) |
|  | 12/9 | 2 (2.2%) |
|  | 9/10 | 2 (2.2%) |
| MAO-A VNTR | 4/4 | 17 (18.9%) |
|  | 4/3 | 36 (40.0%) |
|  | 3/3 | 8 (8.9%) |
|  | 3.5/4 | 1 (1.1%) |
|  | 3.5/3.5 | 2 (2.2%) |
|  | 3 | 10 (11.1%) |
|  | 4 | 14 (15.5%) |
|  | 5 | 2 (2.2%) |

*MAO-A being located on chromosome X, male participants comport only one allele of the gene. All results were concordant with participants' gender. VNTR: Variable Number Tandem Repeats – 5-HTTLPR: Serotonin Transporter-Linked Polymorphic Region – S: Short – L: Long - a: adenine – g: guanine*

Table 4

*Average impulsivity scores per gene and phenotypes*. *Results are presented as Mean ±SD.* *Significant results are underlined in green. Results' ranks are 1 to 5 (Likert scale with 5 levels). High scores refer to high impulsivity.*

| **Genes** | **Phenotypes** | **Pervasive Influence of Feelings**  **T0** | **Lack of Follow Through**  **T0** | **Feelings Trigger Action**  **T0** | **Feelings Trigger Action**  *Screening* |
| --- | --- | --- | --- | --- | --- |
| 5HTTLPR and rs25531 | Low | 3.32±.16 | 2.94±.13 | 3.31±.09 | 3.43±.06 |
|  | Moderate | 3.11±.12 | 2.69±.98 | 3.01±.07 | 3.12±.05 |
|  | High | 3.19±.21 | 2.83±.17 | 3.02±.13 | 3.01±.08 |
| STin2 VNTR | Low | 3.26±.22 | 3.03±.17 | 3.19±.13 | 3.32±.09 |
|  | Moderate | 3.22±.14 | 2.84±.11 | 3.11±.09 | 3.18±.06 |
|  | High | 3.13±.14 | 2.65±.11 | 3.06±.09 | 3.20±.06 |
| MAO-A VNTR | Low | 3.28±.12 | 2.75±.11 | 3.23±.08 | 3.27±.05 |
|  | High | 3.08±.14 | 2.85±.10 | 2.97±.08 | 3.12±.06 |

*All analyses were controlled for age and gender. VNTR: Variable Number Tandem Repeats – 5-HTTLPR: Serotonin Transporter-Linked Polymorphic Region*

Table 5

*ANCOVA results of impulsivity factor levels (see Table 4) differences per polymorphism phenotype (controlled for age and gender)*

| *Genes* | **Pervasive Influence of Feelings**  *T0* | **Lack of Follow Through**  *T0* | **Feelings Trigger Action**  *T0* | **Feelings Trigger Action**  *Screening* |
| --- | --- | --- | --- | --- |
| 5HTTLPR and rs25531 | *F*(2,62)=.563, *p*=.572, *np^2^*=.018 | *F*(2,62)=1.238, *p*=.297, *np^2^*=.038 | ***F*(2,62)=3.220, *p<.*050, *np^2^*=.094** | ***F*(2,85)=8.853, *p<.*001, *np^2^*=.172** |
| STin2 VNTR | *F*(2,62)=.153, *p*=.859, *np^2^*=.005 | *F*(2,62)=2.249, *p*=.114, *np^2^*=.068 | *F*(2,62)=.299, *p=*.743*, np^2^=*.010 | *F*(2,85)=.887, *p*=.416, *np^2^*=.020 |
| MAO-A VNTR | F(1,63)=.957, *p*=.332, *np^2^*=.015 | *F*(1,62)=.332, *p*=.567, *np^2^*=.005 | ***F*(1,63)=4.927, *p<.*050, *np^2^=*.073** | *F*(1,86)=3.620, *p*=.060, *np^2^*=.040 |

*VNTR: Variable Number Tandem Repeats – 5-HTTLPR: Serotonin Transporter-Linked Polymorphic Region*

1. **EEG methods**

The electroencephalogram (EEG) was recorded from 64 electrodes using a BIOSEMI Active-Two system (BioSemi, Amsterdam, The Netherlands; channels Fp1, AF7, AF3, F1, F3, F5, F7, FT7, FC5, FC3, FC1, C1, C3, C5, T7, TP7, CP5, CP3, CP1, P1, P3, P5, P7, P9, PO7, PO3, O1, Iz, Oz, POz, Pz, CPz, Fpz, Fp2, AF8, AF4, AFz, Fz, F2, F4, F6, F8, FT8, FC6, FC4, FC2, FCz, Cz, C2, C4, C6, T8, TP8, CP6, CP4, CP2, P2, P4, P6, P8, P10, PO8, PO4, O2 as well as the left and right mastoid, relative to common mode sense (CMS) and driven right leg (DRL) electrodes).

Based on the literature, asymmetry scores (AS) were computed for the frontal and prefrontal regions. The left frontal region included the following electrodes: Fp1|AF7|AF3|F1|FT7|FC5|FC1|F3|F5|F7|FC3. The right frontal region included the following electrodes: Fp2|AF8|AF4|F2|FT8|FC6|FC2|F4|F6|F8|FC4. The left prefrontal region included the following electrodes: Fp1|AF7|AF3|F1|F3|F5|F7. The right prefrontal region included the following electrodes: Fp2|AF8|AF4|F2|F4|F6|F8.

As suggested in previous research, the alpha asymmetry was indexed using laterality coefficients (LC) using the following formula: LC= (power right – power left)/(power right + power left) * 100. Values superior to zero indicate higher alpha activity in the right cortex compared to the left one, in other words, a greater left cortical activity.

1. **Additional information about NoSTRESS**

Maybe some of you have already read articles about NoSTRESS (e.g., Javelle et al., 2021) and may wonder why N=90 at screening (and not 91) and N=67 at T0 (and not 66) in this manuscript. Out of all blood samples gathered at screening, the quality of the extracted DNA from one participant was not sufficient to perform the polymorphism analysis bringing the N from 91 to 90 (see Figure 2). The randomization for the study NoSTRESS was performed after T0 and included 66 participants (see Javelle et al., 2021). Nonetheless, one participant dropped out during the T0 testing (but after performing all analyses included in this manuscript) and thus before the randomization. Therefore, 66 participants were randomized for the study NoSTRESS, but 67 performed all the tests relevant to our manuscript.
